# Les facteurs prédictifs de morbimortalité chez les patients sous ballon de contre pulsion intra-aortique en chirurgie cardiaque

**DOI:** 10.11604/pamj.2015.21.310.6382

**Published:** 2015-08-28

**Authors:** Brahim Elahmadi, Youssef Motiaa, Abdedaim Elghadbane Hatim, Noureddine Atmani, Younes Moutakillah, Fouad Amal Wahid, Youssef Elbekkali, Mahdi Ait Houssa, Rachid Razine, Abdelatif Boulahya, Mohammed Drissi

**Affiliations:** 1 Réanimation de Chirurgie Cardiaque, Hôpital Militaire d'instruction Mohammed V, Faculté de Médecine et de Pharmacie, Université Mohammed V, Souissi, Rabat, Maroc; 2 Service de Chirurgie Cardiaque, Hôpital Militaire d'Instruction Mohammed V, Faculté de Médecine et de Pharmacie de Rabat, Université Mohammed V, Souissi, Rabat, Maroc; 3 Laboratoire d’épidémiologie et de recherche clinique, Faculté de Médecine et de Pharmacie, Université Mohammed V, Souissi, Rabat, Maroc

**Keywords:** Ballon de contre-pulsion intra-aortique, chirurgie cardiaque, mortalité, morbidité, intra-aortic counterpulsation balloon, cardiac surgery, mortality, morbidity

## Abstract

Le ballon de contre pulsion intra-aortique (BCPIA) est fréquemment utilisé en chirurgie cardiaque, comme moyen d'assistance circulatoire en cas de bas débit cardiaque. Il est d'intérêt clinique de déterminer les facteurs pronostiques chez les patients porteurs d'un BCPIA en chirurgie cardiaque, et qui restent un sujet rarement élucidé dans la littérature. L'objectif de notre travail est de déterminer les facteurs prédictifs de morbi-mortalité chez les patients sous ballon de contre pulsion intraortique en périopératoire d'une chirurgie cardiaque. Il s'agit d'une étude rétrospective portant sur l'ensemble des patients opérés en chirurgie cardiaque sous circulation extracorporelle, et ayant bénéficiés de la mise en place d'un ballon de contre pulsion intra-aortique en périopératoire, au service de chirurgie cardiovasculaire de l'Hôpital Militaire Mohamed V de Rabat, entre le mois de janvier 2005 et le mois d'aout 20014. Soixante dix patients ont été inclus dans notre étude. En analyse univariée l'âge, la dyspnée de stade III et IV, l'insuffisance cardiaque, la présence d'un infarctus du myocarde, d'une coronaropathie mono et bitronculaire, les anomalies du doppler de trons supra-aortique et du membre inférieur, le caractère urgent de la chirurgie, la durée de la circulation extracorporelle, l'instabilité hémodynamique postopératoire, le saignement et l'insuffisance rénale postopératoire étaient statistiquement associés à une mortalité postopératoire élevée. La dyskinésie préopératoire et la sortie de circulation extracorporelle sous drogues étaient associées à une morbidité globale élevée. En analyse multi variée, seule l'âge, constituait un facteur de risque indépendant de mortalité dans notre série avec un Odds Ratio (OR): 1,89 ; un Intervalle de Confiance (IC) 95% de (1,52-4,97) et un p =0,045. Au terme de notre étude, le taux de mortalité était de 48,57% et de morbidité globale était de 87,1%. Il nous parait donc nécessaire pour diminuer l'incidence de cette morbimortalité dans notre population, d'agir sur les facteurs que nous jugeons modifiables tels l'amélioration de la fonction cardiaque préopératoire, l'optimisation de la fonction rénale, la réduction de la durée de CEC et le contrôle du saignement.

## Introduction

Le BCPIA est fréquemment utilisé en chirurgie cardiaque comme moyen d'assistance hémodynamique, Son utilisation est facilitée par la disponibilité de cathéters de plus en plus fins ainsi que par la pose percutanée par la méthode Sildinger [1, 2]. Cependant, ce n'est pas réellement un système d'assistance circulatoire car il n'assure pas un débit autonome. Le principe repose sur le gonflement rapide par un gaz à inertie faible d'un ballonnet placé dans l'aorte descendante. L'inflation est synchronisée sur l'électrocardiogramme durant la diastole améliorant ainsi le flux phasique de la circulation coronaire, la déflation rapide juste avant la systole diminue le travail d'éjection du ventricule gauche [3]. Le BCPIA est parfois associé à des complications graves qui pourraient contrebalancer ses effets hémodynamiques bénéfiques potentiels. Par conséquent, il est d'intérêt clinique de déterminer les facteurs pronostiques chez les patients qui portent un BCPIA en chirurgie cardiaque, et qui reste un sujet rarement élucidé dans la littérature comparativement à son intérêt dans la prise en charge des coronaropathies et dans la salle de cathétérisme.

## Méthodes

Il s´agit d´une étude rétrospective qui a porté sur l'ensemble des patients opérés en chirurgie cardiaque sous circulation extracorporelle, pour un remplacement valvulaire ou un pontage aorto-coronaire, et ayant bénéficiés de la mise en place d'un ballon de contre pulsion intra-aortique en périopératoire, au service de chirurgie cardiovasculaire de l'Hôpital Militaire Mohamed V de Rabat, sur une période du mois de janvier 2005 au mois d'aout 2014. Plusieurs paramètres ont été recueillis: l´âge, le sexe, les antécédents médicaux et chirurgicaux, les résultats du bilan préopératoire, le déroulement de l´acte opératoire et l´évolution postopératoire. L´exploration préopératoire comportait systématiquement: un électrocardiogramme, une radiographie thoracique, une échocardiographie, une échographie des troncs supra-aortiques, une coronarographie, un écho-doppler des membres inferieurs, un bilan biologique préopératoire, et un bilan infectieux. Tous les malades ont bénéficié d´une anesthésie générale associant l'étomidate à la dose de 0,3 mg/kg, le fentanylà la dose de 5 'g /kg et un curare de type cisatracurium à la dose de 0,15 mg/kg ou du rocuronuim à la dose de 0,6 mg/Kg pour l´induction, puis sevoflurane, propofol et morphinique pour l´entretien, l´incision était une sternotomie médiane. Le monitorage hémodynamique peropératoire était basé sur l'enregistrement de rythme cardiaque avec analyse du segment ST, la mesure de pression artérielle invasive et la pression veineuse centrale, la saturation pulsée en oxygène, la température centrale, la diurèse, le temps de coagulation activée et la gazométrie artérielle.

La ventilation artificielle per et postopératoire était standardisée pour tous les patients, avec un volume courant de 6 à 8ml/kg sans dépasser une pression de plateau de 30 cm H2O et en fonction de la capnographie, une fréquence respiratoire à 12 cycles/min et une pression expiratoire positive ne dépassant pas 5cm H2O. La circulation extracorporelle était installée entre une canule aortique et canule veineuse atrio-cave après un bolus d'héparine à la dose de 300UI/kg avec un objectif de temps de coagulation activée à 400 secondes; La circulation extracorporelle est conduite en hémodilution totale avec une hypothermie modérée à 32°C ; Apres clampage aortique, la protection myocardique était assurée par une cardioplégie cristalloïde froide intermittente antérograde administrée à la racine de l'aorte jusqu'à l'arrêt cardiaque puis répétée toutes les 25 à 30 minutes et le refroidissement local par une solution glacée coulée dans le péricarde. La surveillance per circulation extracorporelle avait comme objectif de maintenir : un taux d'hématocrite entre 25 et 30%, une pression artérielle moyenne entre 50 et 80 mmHg, une SpO2 entre 98 et 100%, une SvO2 ' 70% mesurée dans le circuit veineux de la CEC, une PaO2 > 100 mmHg, une PaCO2 < 40 mmHg, et une glycémie entre 1,1 et 1,5g/l. La sortie de la circulation extracorporelle était après réchauffement lent du patient pour atteindre une température centrale '35 °, le recours à l'introduction de Dobutamine, de Noradrénaline et/ ou d'adrénaline était en fonction de l'état hémodynamique et de la contractilité cardiaque. L'héparine était antagonisée par une dose initiale de sulfate de protamine à la dose de 1mg pour chaque 100 UI d'héparine administrée. Une deuxième dose de 0,5 à 1 mg /kg de sulfate de protamine était administrée si l'ACT restait supérieure à 140 secondes. La prévention du saignement était assurée par l'administration de l'acide tranexamique à la dose de 15 à 30 mg/kg en deux prises avant l'injection de l'héparine et avant l'injection de la protamine.

Les définitions suivantes ont été retenues pour notre étude : L'Infarctus du myocarde répondait à la définition universelle [4]. Le dosage de la Troponine I a été systématiquement réalisé en postopératoire, ou immédiatement en cas d´instabilité hémodynamique et de troubles de la repolarisation ou du rythme sur l´électro-cardiogarmme, puis chaque jour jusqu´à la normalisation. L´insuffisance rénale aigue répondait à la classification de RIFLE [5]. Les complications respiratoires postopératoires majeures ont été définies par l´existence de l´une ou plus des situations suivantes: une difficulté de sevrage de la ventilation artificielle après plus de 48 heures, la nécessité d´une reventilation invasive ou non invasive, et l´apparition d´un syndrome de détresse respiratoire aiguë, d´une pneumopathie infectieuse ou d´une macro-atélectasie [6]. La Morbidité neurologique est définie par la survenue d'une aphasie, d'un trouble de comportement, ou d'un déficit moteur confirmé à l'examen clinique ou au scanner cérébral. La Morbidité infectieuse est définie par la présence d'une preuve bactériologique d'une infection pulmonaire ou urinaire, d'une médiastinite, d'une infection du site opératoire, ou au niveau du point d'insertion du BCPIA. Le saignement postopératoire est évalué par un volume de liquide recueilli par les drains supérieur à 100cc/h. Une thrombopénie est retenue pour un taux de plaquettes inférieur à 150000 /mm^3^. Les Complications digestives étaient définies par la survenue d'une hémorragie digestive, d'un ulcère gastrique, d'une pancréatite ou d'une cholécystite alithiasique. Les anomalies du doppler des troncs supraortiques sont définies par la présence d'une lésion athéromateuse. Les anomalies du doppler du MI sont définies par la présence d'une thrombose veineuse profonde. La Morbidité globale était définie par la survenue d'une ou de plusieurs des morbidités sus-citées. La Mortalité opératoire était définie par tout décès survenu dans les 30 jours suivant l'opération.

Notre critère de jugement principal était la survenue de toute morbi-mortalité périopératoire. L´analyse statistique a été réalisée à l´aide du logiciel SPSS 13.0. Les variables quantitatives ont été exprimées en moyennes± déviations standard ou médiane et quartiles en fonction de la distribution des variables, et les variables qualitatives en effectifs et pourcentages. Les variables quantitatives ont été comparées par le test t de Student ou le test U de Mann-Whitney, et les variables qualitatives ont été comparées à l´aide du test de Chi 2 de Pearson ou du test exact de Fisher. Le degré de significativité a été fixé un p < 0,05. L´analyse multivariée a fait appel à la régression logistique sur un modèle incluant les paramètres dont le p était inférieur ou égal à 0,2 en analyse univariée.

## Résultats

Soixante dix patients parmi 1903 ont été inclus dans notre étude, seize femmes (22,9%) et cinquante quatre hommes (77,1%). L'âge médian était de 57 (50,75 - 65). Trente patients (42,8%) avaient présenté un infarctus du myocarde en préopératoire, la dyspnée stade III et IV était constaté chez 41 patients (58,57%), 22 patients (31,42%) étaient en insuffisance cardiaque. Les caractéristiques épidémiologiques, cliniques et par-acliniques préopératoires sont représentées dans le Tableau 1. Les données per et postopératoires sont résumées dans le Tableau 2. Dans notre série le BCPIA a été inséré chez 68 patients (97,14%) en postopératoire. Cinquante sept patients (81,42%) ont présenté une instabilité hémodynamique en postopératoire, avec un infarctus du myocarde chez 17 patients (24,28%) et des troubles de rythmes chez 25 patients (35,71%). Les complications respiratoires postopératoires ont été observé chez 17 patients (24,29%), la une nécessité de ventilation artificielle prolongée était chez 52 patients (85,26%). Trente trois patients (47,14%) ont présenté une insuffisance rénale postopératoire. Le taux de mortalité était de 48,57%. La comparaison des principaux éléments cliniques, paracliniques, et évolutifs entre le groupe des patients survivants (groupe S) et celui des patients décédés (groupe D), figure sur le Tableau 3. La présence d'une dyspnée de stade III et IV, d'une insuffisance cardiaque, d'une coronaropathie monotronculaire, d'une chirurgie urgente, d'une instabilité hémodynamique postopératoire et la survenue d'une insuffisance rénale aigue postopératoire étaient plus important dans le groupe D avec une différence statistiquement significative. En analyse univariée qui figure dans les Tableau 4 et Tableau 5: l'âge, la dyspnée, l'insuffisance cardiaque, la présence d'un infarctus du myocarde, d'une coronaropathie monotronculaire et bitronculaire, les anomalies du doppler des troncs supra-aortiques et du membre inférieur, le caractère urgent de la chirurgie, la durée de la circulation extra-corporelle, l'instabilité hémodynamique postopératoire, le saignement et l'insuffisance rénale postopératoire étaient statistiquement associés à une mortalité postopératoire plus élevée. La dyskinésie préopératoire et la sortie de circulation extra-corporelle sous drogues étaient associées statistiquement à une morbidité globale élevée. En analyse multi variée, seule l'âge constituait un facteur de risque indépendant de mortalité dans notre série avec un Odds Ratio (OR): 1,89 ; un Intervalle de Confiance (IC) 95% de [1,52-4,97] et un p =0,045. En analyse multivariée aucune association avec la morbidité globale n'a été retrouvée de façon statistiquement significative.

**Tableau 1:** les données préopératoires

Caractéristiques	*N= 70*
**Age (année)**	57 [50,75 – 65]
**Sexe**	
Home	54 (77,1%)
Femme	16(22,9%)
**Hypertension artérielle**	15 (22,4%)
**Tabagisme**	34 (48,6%)
**Diabète**	23 (32,9%)
**IDM**	
Récent (moins de 3 mois)	22 (31,4%)
Ancien (plus de 3 mois)	6 (8,6%)
Stenté	2 (2,9%)
**Broncho-pneumopathie chronique obstructive**	3(4,3%)
**Insuffisance Rénale**	
Sans dialyse	1(1,4%)
Avec dialyse	1(1,4%)
**Artériopathie**	8(11,4%)
**Endocardite**	1(1,4%)
**Dyspnée (NYHA)**	
Stade I	5(5,1%)
Stade II	20(28,6%)
Stade III	27(38,6%)
Stade IV	14(20%)
**Insuffisance cardiaque**	
Gauche	15(21,4%)
Droite	4(5,7%)
Globale	3(4,3%)
**Anomalie radiologique**	50/ 71,4%
**ECG**	
Troubles de rythme	21(30%)
Troubles de repolarisation	33(47,1%)
**Fraction d’éjection du ventricule gauche**	45 [34,5 – 55,75]
**Dyskinésie**	48(40%)
**Coronaropathie**	
Monotronculaire	8(11, 43%)
Bitronculaire	6(8, 57%)
Tritronculaire	26(37, 14%)
**Anomalies de Doppler du Tronc supraaortique**	9(12,86%)
**Anomlaies de Doppler du membre inférieur**	11(15,71%)
**Hémoglobine**	13[11- 14]
**Plaquettes**	212000 [182000 – 245000]
**Euroscore**	
< 6	41 (59,4%)
≤ 6	29 (40,6%)

**Tableau 2:** les données per et postopératoires

Variables	Valeurs
**Inotrpes avant induction**	23(32,85%)
**Instabilité hémodynamique préopératoire**	13 (18,57%)
**BCPIA avant induction**	2 (2,86%)
**Chirurgie programmée**	54(77,14%)
**Durée du clampage aortique (min)**	82[68,75 – 105]
**Durée de CEC (min)**	135[116 – 167,25]
**Drogues à la sortie de CEC**	66(94,28%)
**Durée de chirurgie (min)**	300[240- 360]
**Transfusion**	34(48,58%)
**Instabilité hémodynamique post opératoire**	57(81,42%)
**BCPIA postopératoire**	68(97,15%)
Complications respiratoires postopératoires	17(24,29%)
Ventilation artificielle prolongée	52(85,26%)
Réintubation	12(17,14%)
**IDM postopératoire**	17(24,28%)
**Troubles de rythme**	25(35,71%)
**IRA postopéraoire**	33(47,14%)
**Dialyse postopératoire**	6(8,57%)
**Transfusion postopératoire**	39(55,71%)
**Saignement(ml)**	550[350- 1100]
**Reprise chirurgicale**	7(10%)
**Acidose lactique**	51(72,85%)
**Infection**	
pulmonaire	20(28,57%)
paroi	1(1,43%)
médiastinite	1(1,43%)
**Thrombopénie**	58(82,85%)
**Troubles neurologiques**	6(8,57%)
**Complications digestives**	4(5,71%)
**Séjour en reanimation(jours)**	7[4-13]
**Décès**	34(48,57%)

**Tableau 3:** comparaison entre les 2 groupes survivants et décédés

Variables	Malades non décédés (S) 36	Malades décédés (D) n 34	p value
**Age**	60[52,25 – 64,5]	54[47,25 – 66]	0,26
**Sexe**	31/57,4%	23/42,6%	0,45
**HTA**	9/60%	6/40%	0,45
**Tabagisme**	18/52,9%	16/47%	0,8
**Diabète**	12/52,2%	11/47,8%	0,33
**IDM**			
**Récent (moins 3 mois)**	14/63,6%	8/36,4%	0,004
**Ancien (plus 3 mois)**	6/100%	0	
**Stenté**	2/100%	0	
**Bronchopneumopathie**	2/66,7%	1/33,3%	0,6
**Insuffisance Rénale**			0,34
**Sans dialyse**	0	1/100%	
**Avec dialyse**	0	1/100%	
**Artériopathie**	5/62,5%	3/37,5%	0,7
**Endocardite**	0	1/100%	0,49
**Dyspnée**			0,03
Absente	4/100%	0	
Stade I	4/80%	1/20%	
Stade II	15/75%	5/25%	
Stade III	9/33,3%	18/66,7%	
Stade IV	4/28,6%	10/71,4%	
**Insuffisance cardiaque**			0,035
Gauche	5/33,3%	10/66,7%	
Droite	1/25%	3/75%	
Globale	0	3/100%	
**Fraction d’éjection de VG**	42[30,75 – 55]	46[35- 58,25]	0,6
**Dyskinésie**	18/64,3%	10/35,7%	0,08
**Coronaropathie**			0,01
Absente	8/28,6%	20/71,4%	
Monotronculaire	2/25%	6/75%	
Bitronculaire	6/75%	2/25%	
Tritronculaire	20/76,9%	6/23,1%	
**Anomalies du TSA**	10/88,9%	1/11,1	0,03
**Anomlaies du Doppler de MI**	10/90,9%	1/9,1%	0,04
**Hémoglobine**	12,5[11-13]	14[13-14]	0,11
**Plaquettes**	224000[171250-267500]	209000[187000-237000]	0,5
**Instabilité HED préopératoire**	4/30,8%	9/69,2%	0,1
**Drogues préopératoire**	10/43,5%	13/56,5%	0,35
**Chirurgie urgente**	2/13,3%	13/86,7%	0,001
**BCPIA sortie CEC**	23/63,9%	13/36,1%	0,032
**Durée du clampage**	80[64,25 – 104,75]	94,5[72,25 – 130]	0,11
**Durée de CEC**	128,5[108,25 – 153,75]	147,5[117,5 – 180]	0,08
**Durée de chirurgie**	318[241,25 – 360]	272,5[217,5 – 360]	0,3
**Réintubation**	5/41,7%	7/58,3%	0,45
**Complications respiratoires**	6/35,3%	11/64,7%	0,12
**VM prolongée**	25/48,1%	27/51,9%	0,73
**Instabilité HED postopératoire**	24/42,1%	33/57,9%	0,001
**BCPIA en post opératoire**	35/51,5%	33/48,5%	0,96
**IDM post opératoire**	7/41,2%	10/58,8%	0,33
**Troubles de rythme**	12/48%	13/52%	0,7
**IRA postopératoire**	11/33,3%	22/66,7%	0,004
**Transfusion postopératoire**	23/59%	16/41%	0,16
**Reprise chirurgicale**	4/57,1%	3/42,8%	0,1
**Acidose lactique**	24/47,1%	27/52,9%	0,23
**infection**	14/60,9%	9/39,1%	0,27
**Thrombopénie**	30/51,7%	6/48,3%	0,91
**Troubles neurologiques**	5/88,3%	1/16,7%	0,12
**Complications digestives**	0	4/100%	0,05
**Ischémie de MI**	3/60%	2/40%	0,7
**Morbidité globale**	28/45,9%	33/54,1%	0,02
**Séjour en réanimation**	6[3,25 – 12,75]	8,5[4- 13,5]	0,32

**Tableau 4:** analyse univariée de la mortalité

variable	mortalité		
	OR	P	IC
**Age**	1,5	0,038	1,2-2,5
**Sexe**	0,27	0,07	0,08-0,99
**HTA**	0,64	0,45	0,19-1,98
**Tabagisme**	0,9	0,8	0,29-1,93
**Diabète**	0,9	0,93	0,32-2,54
**IDM**	0,22	0,01	0,21-0,77
**BPCO**	0,51	0,6	0,13-7,76
**Artériopathie**	0,6	0,5	0,12-0,89
**euroscore**	1,63	0,32	0,61-4,31
**Dyspnée**	2,97	0,01	1,23-12,45
**Insuffisance cardiaque**	3	0,01	1,66-15,04
**Fractio d’éjection du VG**	1	0,6	0,98-1,04
**Dyskinésie**	0,41	0,08	0,14-1,06
**Coronaropathie**	0,49	< 0,001	0,32-0,73
**Anomalies du TSA**	0,1	0,04	0,01-0,63
**Anomlaies de Doppler de MI**	0,07	0,01	0,1-0,68
**Hémoglobine**	2,6	0,2	0,57-9,65
**Plaquettes**	1	0,7	1-1
**BCPIA en préopératoire**	0,27	0,01	0,1-0,74
**Instabilité HED pré opératoire**	2,79	0,12	0,77-10,13
**Chirurgie urgente**	10,5	0,004	2,09-49,82
**Durée du clampage**	1,016	0,02	1,002-1,031
**Durée de CEC**	1	0,04	1-1,21
**Durée de chirurgie**	1	0,4	0,99-1,003
**Drogues à la sortie de CEC**	3	0,4	0,60-49,78
**BCPIA à la sortie de CEC**	0,35	0,03	0,121-0,863
**Réintubation**	1,6	0,5	0,44-5,48
**VM prolongée**	1,35	0,07	0,51-4,32
**Index d'oxygénation**			
300- 200	1		
200-100	17	0,009	2,02-142,84
<100	6204	0,77	0,00-3,3^E^+29
**Instabilité HED post opératoire**	16,5	0,009	2,09-141,75
**BCPIA en post opératoire**	1	0,94	0,06-16,16
**IDM post opératoire**	0,72	0,33	0,55-5,05
**Troubles de rythmes**	1,23	0,7	0,68-1,68
**IRA post opératoire**	4,16	0,005	1,46-10,89
**Transfusion post opértaoire**	0,5	0,15	0,21-4,65
**Reprise chirurgicale**	0,77	0,75	0,23-5,64
**Saignement post opératoire**	1	0,007	0,09-2,56
**Acidose lactique**	1,92	0,7	0,68-5,95
**Thrombopénie**	0,93	0,91	0,23-2,40
**Troubles neurologiques**	0,18	0,13	0,01-2,01
**Ischémie de MI**	0,6	0,7	0,10-4,26
**Séjour en réanimation**	1	0,89	0,942-1,058

**Tableau 5:** analyse univariée de la morbidité globale

variable	Morbidité globale		
	OR	P	IC
**Age**	1,02	0,42	0,67-2,05
**Sexe**	1,04	0,96	0,45-1,67
**HTA**	0,42	0,43	0,24-1,56
**Tabagisme**	0,48	0,33	0,14-1,76
**Diabète**	1,02	0,96	0,85-1,65
**IDM**	1,43	0,4	0,89-2,04
**Bronchopneumopathies**	3,69	0,3	0,57-5,78
**Artériopathie**	0,54	0,83	0,23-1,89
**euroscore**	1,89	0,67	0,76-5,72
**Dyspnée**	0 ,37	0,45	0,25-1,56
**Insuffisance cardiaque**	2,3	0,08	0,89-3,56
**Fraction d’éjection du VG**	1	0,86	0,62-2,04
**Dyskinésie**	3,54	0,009	1,95-6,87
**Coronaropathie**	0,35	0,81	0,29-2,06
**Anomalies du TSA**	2,22	0,37	0,86-3,08
**Anomlaies du Doppler de MI**	1,65	0,57	0,45-5,81
**Hémoglobine**	1,29	0,61	0,29-4,86
**PLaquettes**	1,67	0,78	0,45-6,72
**BCPIA en préopératoire**	0,5	0,45	0,18-2,85
**Instabilité HED en pré opératoire**	0,42	0,43	0,26-3,81
**Chirurgie urgente**	3,30	0,11	0,71-7,48
**Durée de clampage**	1	0,75	0,63-2,75
**Durée de CEC**	1	0,3	0,72-4,36
**Durée de chirurgie**	1	0,24	0,63-3,08
**Drogues à la sortie de CEC**	0,12	0,048	0,08-2,89
**BCPIA à la sortie de CEC**	3,89	0,1	0,46-8,79

## Discussion

L´incidence de l'insertion du ballon de contre pulsion intraortique, en chirurgie cardiaque reste très diversement appréciée dans les différentes études selon les critères retenus pour l'assistance circulatoire, ce qui la fait varier de 1,5 à plus de 27% [7, 8], alors que dans notre étude, elle est 3,7%. L´incidence de la mortalité chez les patients sous BCPIA était de 48,57% ce qui reste n'est pas élevé par rapport aux études publiées [7, 9, 10]; et celle de La morbidité globale était de 87,1% avec une prédominance de sa survenue dans le groupe D.

L´âge avancé est fréquemment discuté dans la littérature comme facteur de risque de morbimortalité chez les patients sous BCPIA. On considère souvent qu´il n´est pas un facteur de risque indépendant si l´on tient compte des comorbidités qui y sont associées. Cependant, certaines études bien menées suggèrent un rôle propre de l´âge, dès 65 ans et le risque augmente surtout après 70 ans [9, 11]. Dans notre étude, l'âge médian des patients dans le groupe D est de 54[47,25-66] et selon l'analyse multi variée seul l'âge est un facteur indépendant de mortalité avec un OR: 1,89 ; IC 95%: (1,52-4,97) ; p: 0,045.

Dans notre étude, et en phase préopératoire la présence d'un infarctus du myocarde récent, d'une coronaropathie, d'une dyspnée de stade III ou IV, et d'une insuffisance cardiaque étaient significativement associés à une mortalité élevée avec un p < 0,05; par contre on trouve que les anomalies détectées au doppler du membre inférieur et du tronc supra-aortique étaient plus élevées dans le groupe des survivants avec une différence significative. D´autres facteurs de risques de mortalité sont rapportés dans la littérature tels que la présence d'un angor instable, de sexe féminin, d'un stade avancé de NYHA, d'une fraction d'éjection de ventricule gauche basse inférieure à 40%, d'une atteinte tric-tronculaire ou du tronc coronaire gauche [11, 12]. Karimi et al [13], ont identifié l'insuffisance cardiaque gauche, et le diabète comme facteurs de risque indépendants de mortalité chez les patients opérés pour pontage coronaire et ayant bénéficiés d'un BCPIA.

Le caractère urgent de la chirurgie est identifié comme facteur de risque de mortalité dans la littérature [11], ce qui rejoint le résultat de notre étude, ceci s´explique à notre sens par le fait que la chirurgie urgente survient sur une fonction cardiaque perturbée avec un état hémodynamique altérée. Khan et al, ont publié le rôle bénéfique de l'insertion du BCPIA en préopératoire d'une chirurgie urgente chez les patients souffrant d'un infarctus du myocarde récent [14].

La durée de la circulation extracorporelle, l'insertion du BCPIA à la sortie de cette circulation, l'instabilité hémodynamique postopératoire, la survenue d'une insuffisance rénale postopératoire ainsi que le saignement postopératoire sont significativement associés à une mortalité élevée dans notre étude. D´autres études ont démontré que l'arrêt cardiaque, l'insuffisance rénale postopératoire, le bas débit cardiaque, l'infarctus du myocarde et l'insertion du BCPIA en postopératoire sont des facteurs de mortalité dans la chirurgie cardiaque avec BCPIA [11, 13]. Et comme affirme certaine étude, le dépistage et la prévention de l'insuffisance rénale postopératoire sont des facteurs sur lesquels, il est possible d´agir dans le but de prévenir le risque élevé de morbimortalité [15, 16].

Le timing de l'insertion du BCPIA en périopératoire chez les patients à haut risque reste un sujet très controversé [8, 9, 12, 17–19]. Dans notre étude seulement 2 patients avaient un BCPIA en préopératoire sans que la différence ne soit significative. Ota et al [20], et dans une étude comparative sur l'effet du BCPIA en postopératoire de la chirurgie valvulaire et du pontage coronarien. Ils ont démontré que le BCPIA était plus efficace chez les patients avec un pontage aorto-coronnaire par rapport à ceux avec un remplacement valvulaire. Ceci s'explique par la fonction cardiaque perturbée et par l´incidence des complications péri-opératoires graves qui étaient plus élevées chez les patients valvulaires. Dans notre étude le pontage aorto-coronaie représente 53,7% de l'ensemble des indications chirurgicales sans que la différence ne soit significative. Chez les patients sous BCPIA, de rares études ont montré une corrélation entre la chirurgie de remplacement valvulaire et le risque de morbimortalité, en présence d'une endocardite infectieuse, d'une prothèse valvulaire antérieure, d'une chirurgie concomitante, d'un stade avancé de NYHA et d'une insuffisance aortique ou mitrale [21].

Dans notre travail, et dans l'analyse uni-variée seules la dyskinésie et la nécessité des drogues à la sortie de la circulation extra-corporelle sont des facteurs de morbidité globale . D´autres facteurs de risques sont rapportés dans la littérature comme facteurs de risque de complications en chirurgie cardiaque chez les patients sous BCPIA, tels que le sexe féminin, le diabète, les antécédents de tabagisme, l'utilisation des antiplaquettaires en préopératoire, l'insuffisance rénale, la fraction d'éjection basse et le recours aux inotropes dans la période postopératoire [7, 22].

Les limites de l'étude sont: il s'agit d'une étude rétrospective avec un échantillon de patients réduit ; Les résultats seraient pertinents si les patients avaient été répartis selon le type de geste opératoire, le moment de l'insertion du BCPIA. Le pronostic est différent d'un groupe à l'autre d'après abdelnoor [21].

## Conclusion

Au terme de notre étude, le taux de mortalité était de 48,57% et de morbidité globale était de 87,1%. L'âge, l'infarctus du myocarde, la dyspnée de stade III et IV, l'insuffisance cardiaque, la coronaropathie, la chirurgie urgente, la durée de la circulation extra-corporelle, l'insertion du BCPIA à la sortie de cette circulation, l'instabilité hémodynamique postopératoire, l'insuffisance rénale postopératoire et le saignement postopératoire étaient statistiquement associés à une mortalité élevée, mais seule l'âge qu'était un facteur indépendant de mortalité à l'analyse uni-variée. Seulement dyskinésie préopératoire était statistiquement associée à une morbidité globale élevée. Il nous parait donc nécessaire pour diminuer l'incidence de cette morbimortalité dans notre population, d'agir sur les facteurs que nous jugeons modifiables tels l'amélioration de la fonction cardiaque préopératoire, l'optimisation de la fonction rénale, la réduction de la durée de circulation extra-corporelle et le contrôle du saignement.
